# A comparative study on the implementation of deep learning algorithms for detection of hepatic necrosis in toxicity studies

**DOI:** 10.1007/s43188-023-00173-5

**Published:** 2023-04-06

**Authors:** Ji-Hee Hwang, Minyoung Lim, Gyeongjin Han, Heejin Park, Yong-Bum Kim, Jinseok Park, Sang-Yeop Jun, Jaeku Lee, Jae-Woo Cho

**Affiliations:** 1 Toxicologic Pathology Research Group, Department of Advanced Toxicology Research, Korea Institute of Toxicology, Daejeon, 34114 Republic of Korea; 2 Department of Advanced Toxicology Research, Korea Institute of Toxicology, Daejeon, 34114 Republic of Korea; 3 Research & Development Team, LAC Inc, Seoul, 07807 Republic of Korea

**Keywords:** Deep learning, Hepatic necrosis, Histopathology, Image analysis, Toxicology

## Abstract

Deep learning has recently become one of the most popular methods of image analysis. In non-clinical studies, several tissue slides are generated to investigate the toxicity of a test compound. These are converted into digital image data using a slide scanner, which is then studied by researchers to investigate abnormalities, and the deep learning method has been started to adopt in this study. However, comparative studies evaluating different deep learning algorithms for analyzing abnormal lesions are scarce. In this study, we applied three algorithms, SSD, Mask R-CNN, and DeepLabV3^+^, to detect hepatic necrosis in slide images and determine the best deep learning algorithm for analyzing abnormal lesions. We trained each algorithm on 5750 images and 5835 annotations of hepatic necrosis including validation and test, augmented with 500 image tiles of 448 × 448 pixels. Precision, recall, and accuracy were calculated for each algorithm based on the prediction results of 60 test images of 2688 × 2688 pixels. The two segmentation algorithms, DeepLabV3^+^ and Mask R-CNN, showed over 90% of accuracy (0.94 and 0.92, respectively), whereas SSD, an object detection algorithm, showed lower accuracy. The trained DeepLabV3^+^ outperformed all others in recall while also successfully separating hepatic necrosis from other features in the test images. It is important to localize and separate the abnormal lesion of interest from other features to investigate it on a slide level. Therefore, we suggest that segmentation algorithms are more appropriate than object detection algorithms for use in the pathological analysis of images in non-clinical studies.

**Supplementary Information:**

The online version contains supplementary material available at 10.1007/s43188-023-00173-5.

## Introduction

In recent years, artificial intelligence (AI) methods involving the use of convolutional neural networks (CNN), also known as deep learning algorithms, have been applied in various fields. Particularly in computer vision tasks, deep learning methods deconvolute the image content into thousands of prominent features and select or aggregate the most meaningful features to identify the complex characters of the image. This process shows high accuracy in image analysis and has, therefore, been actively applied to fields that use image data, such as medical imaging. Within this application, computational analysis of histopathology has recently shown significant advancement with the introduction of slide scanners. A slide scanner generates a whole-slide image (WSI) by combining multiple captured images of entire tissue sections on the slide. This procedure enabled the transition from classical pathology to digital pathology [1, 2] and has been applied to clinical as well as non-clinical studies. According to the Food and Drug Administration (FDA) guidelines, many tissue slides are generated to assess the toxicity of test compounds in non-clinical studies. For example, when following FDA guidelines in rodent subchronic toxicity tests, over 3,000 tissue slides are produced based on 40 different tissues from 20 animals of each sex in each treatment group, for the control and high-dose groups. [3, 4]. Therefore, several studies have attempted to adapt deep learning methods for a more efficient workflow. Despite their ability to streamline this process, these methods are still not widely used in the discipline of toxicologic histopathology [5].

To date the majority of non-clinical studies utilizing these AI methods employ supervised deep learning algorithms provided with hand-labeled annotations [5]. CNN model architectures can be divided into three categories based on how the model predicts the object in the images. First, classification models mainly classify the image as binary or multi-class outcomes, such as predicting positive/negative class or the presence/absence of a region of interest (ROI). Therefore, they are limited and are unable to provide the exact type and location of the ROI within the image. Second, object detection models analyze the ROI, not only by its classification but also by localizing it in the image or video. The object detection model generally detects the ROI with a bounding box (bbox); thus, it has a more intuitive representation than the classification models. Common object detection models include the region-based convolutional neural network (R-CNN) family, you only look once (YOLO) models [6], single-shot detectors (SSD) [7], and RetinaNet [8]. In non-clinical studies, these models have been applied to detect glomeruli [9], differential ovarian follicles [10], and corpora lutea [11]. Finally, image segmentation models classify each pixel in the ROI so that it not only recognizes the instance and spatial location of the ROI but also distinguishes its precise shape from the background. Segmentation architectures include fully convolution networks [12], U-NET [13], DeepLab [14], and Mask region-based CNN (Mask R-CNN) [15]. Our previous studies have shown the successful implementation of Mask R-CNN to detect and quantify the degree of hepatic fibrosis [16] and hepatic lesions involved in acute hepatic injury [17] at the WSI level. However, the evaluation and comparative examination of Mask R-CNN and other deep learning algorithms are necessary for determining an optimal model for detecting lesions in non-clinical studies.

Hepatic necrosis is one of the critical lesions of acute hepatitis frequently seen in drug-induced liver injury, making it a major concern for drug developers, regulatory authorities, and clinicians [18]. The observable morphological patterns of hepatic necrosis can be categorized as spotty or confluent necrosis [19]. In the case of acetaminophen (APAP) overdose, there is apparent centrilobular hepatic necrosis with other hepatic lesions [20]. For this reason, APAP has been used to induce toxicity in hepatic tissues when developing phytotherapeutic and hepatoprotective pharmaceuticals for ameliorating acute hepatic injury [21]. Hence, proper quantification of hepatic necrosis and its comparison among treatment groups is important when examining the toxic effect of a drug in a non-clinical study. To achieve this goal, investigations are needed to find the optimal model for the effective quantification of hepatic necrosis using various deep learning models.

In this study, we trained SSD, Mask R-CNN, and DeepLabV3^+^ in the task of detecting hepatic necrosis. Each model’s performance was evaluated using accuracy, precision, and recall calculations based on its predictions on large-scale images to investigate the optimal deep learning algorithms for detecting hepatic necrosis in non-clinical studies.

## Materials and methods

### Animal experiments

To induce hepatic necrosis in Sprague–Dawley (SD) rats, we conducted acute oral toxicity tests as explained previously [17]. Male and female SD rats (Crl:CD) were obtained from Orient Bio, Inc. (Gyeonggi, Korea) at 9 weeks old. Animals were allowed to acclimate for 2 days prior to the beginning of the study. Throughout the experiments, the rats were maintained under controlled conditions (23 ± 3 ℃, 30–70% relative humidity, 12 h light/12 h dark cycle of 150–300 lx, 10–20 cycles/h ventilation) and provided a standard rat pellet diet (gamma-ray irradiated; 5053 PMI Nutrition International, San Francisco, CA, USA) ad libitum. The animals had free access to municipal tap water that had been filtered and UV-irradiated. This water was analyzed for specific contaminants every 6 months by the Daejeon Regional Institute of Health and Environment (Daejeon, Korea).

An acute oral toxicity study was performed according to the Korea ministry of food and drug safety (MFDS) Test Guideline 2017–71 [23]. Animals were randomly assigned to the following three groups (*n* = 10 per group, 5 males and 5 females): a control group, a single-dose APAP group, and a repeated-dose APAP group. APAP (A7085, 99.0% purity; Sigma-Aldrich, MO, USA) was administered orally to induce acute liver injury in 10-week-old SD rats using two dosing systems: a single dose of 2,500 mg/kg or a 6-day repeated dose of 1000 mg/kg. Doses of APAP were chosen from previously published reports [24, 25]. Immediately prior to administration, 2500 or 1000 mg of APAP was dissolved in 10 mL of sterile distilled water. The administration was performed at 10 mL/kg per dose. Sterile distilled water was administered as vehicle control. The day of the starting dose was regarded as day 1. Single-dose (including vehicle control) and 6-day repeated-dose animals were euthanized by isoflurane inhalation on days 3 and 7, respectively. Liver tissues were collected in 10% formaldehyde. Hematoxylin and eosin (H&E) staining was performed using the left lateral and median lobes of paraffin-embedded livers, and sections were used for digital archiving. The experiment was approved by the Association for Assessment and Accreditation of Laboratory Animal Care International and the Institutional Animal Care and Use Committee (Approval ID: 20–1-0265). All the animal treatments followed the Guide for the Care and Use of Laboratory Animals for animal care [22].

### Data preparation

Slides of liver sections were prepared by three different research centers (Korea Institute of Toxicology, ChemOn Inc., and Biotoxtech) to account for any variation in staining and sectioning techniques. WSIs of liver sections were scanned using an Aperio ScanScope XT (Leica Biosystems, Buffalo Grove, IL, USA) with a 20 × objective lens and bright-field illumination. The scan resolution was 0.5 μm per pixel, and the images were saved as TIFF strips with JPEG2000 image compression. The data preparation for necrosis was performed as previously described [16]. Next, the 20 × magnified WSIs were cropped into 448 × 448-pixel tiles. A total of 500 image tiles were obtained from 14 WSIs, which showed hepatic necrosis among the selected 193 WSIs. All lesions on the acquired image tiles were labeled using a VGG image annotator 2.0.1.0 (Visual Geometry Group, Oxford University, Oxford, UK), with 510 annotations per 500 tiles. The lesions were characterized using nuclear dissolution and fragmentation with pale eosinophilic cytoplasm in the image and hemorrhage (Online Resource 1). These annotations were confirmed by an accredited toxicologic pathologist before the algorithm training was initiated. The lesions identified in these images were labeled and used to train and test the algorithms. The train_test split function embedded into the scikit-learn package was used to split the annotated image tiles into training, validation, and test datasets (ratio of 7:2:1, respectively). Data augmentation, to improve the training dataset, was performed 16 times using a combination of image-augmenting techniques (reverse, rotation, and brightness). The total number of images and annotations used for training, validation, and testing were 5,600, 100, and 50 and 5,680, 104, and 51, respectively (Online Resource 2).

### Training of algorithms and metrics for model performance

#### Model structure

**Fig. 1:**
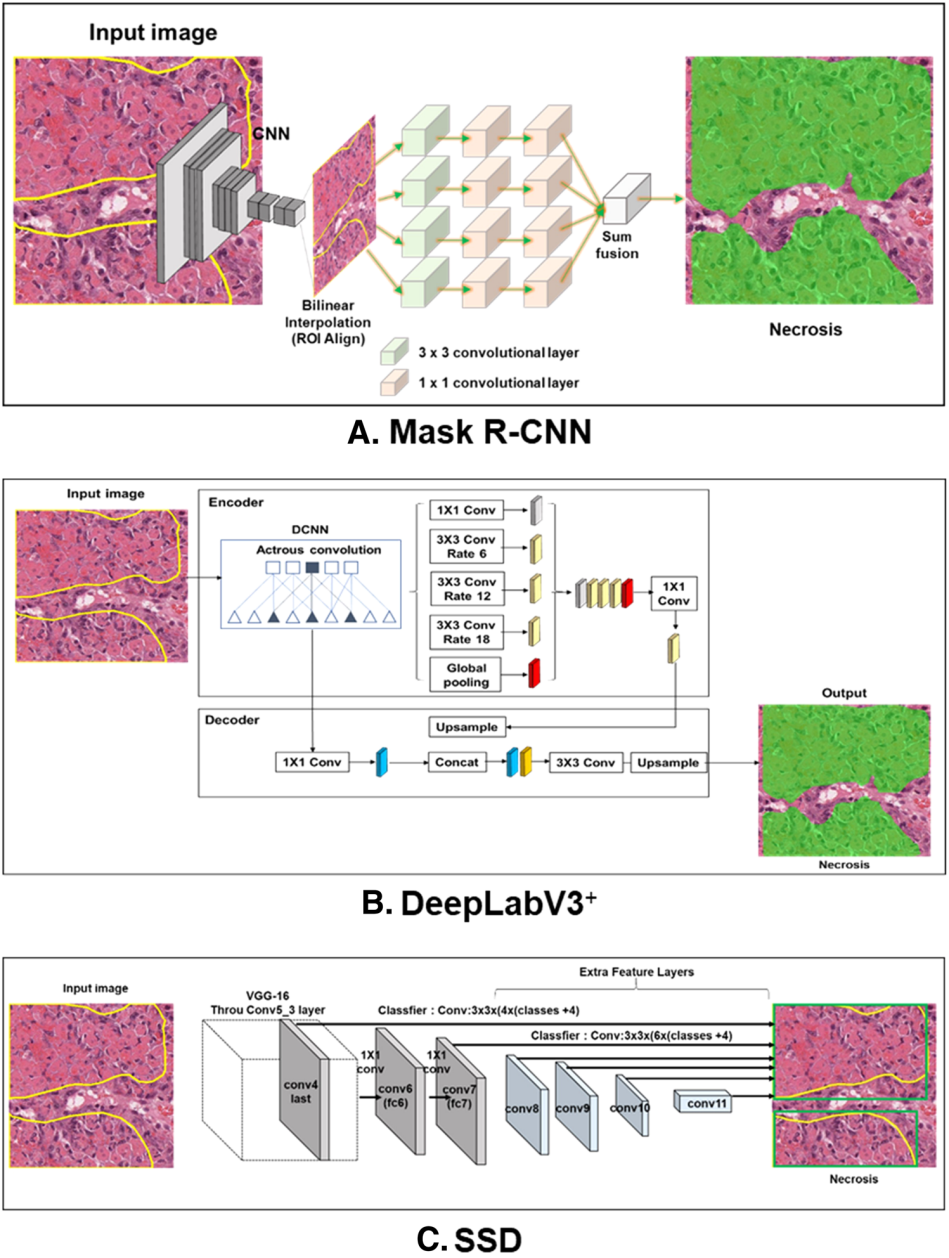
Structures of deep learning networks used in this study. The structure of Mask R-CNN (**a**), DeepLabV3^+^ (**b**), and SSD (**c**)

Three algorithms that have demonstrated great performance in recognizing the object of interest in images in various ways were trained (Fig. 1). Mask R-CNN (Fig. 1a), an instance segmentation model, was developed from Faster R-CNN. It is one of the best-known detection-based segmentation models and uses an ROI alignment (ROI align) with bilinear interpolation to increase the number of anchors and mask branches needed to achieve instance segmentation [15]. DeepLabV3^+^ (Fig. 1b) is a semantic segmentation model that uses the Xception model and applies the depth-wise separable convolution to both Atrous Spatial Pyramid Pooling and decoder modules. Atrous Spatial Pyramid Pooling controls the resolution of features computed by the network by adjusting the field-of-view of the filter to capture multiscale information, allowing the network to explicitly generalize standard convolution operations. Therefore, DeepLabV3^+^ is a faster and stronger encoder-decoder network [14]. Finally, SSD (Fig. 1c), an object detection model, has a base network of VGG16 and an additional auxiliary network. When connecting the two networks, the detection speed is improved by replacing the fully connected layer with a convolutional layer. The SSD model includes a feature map obtained from the convolution layer in the middle of the convolution network and uses a total of six different scale feature maps for prediction. Moreover, for each cell in the feature map, the position of the object is estimated using the default box, which is a bbox with a different scale and aspect ratio. According to this procedure, the SSD has high speed and accuracy as a 1-stage detector with an integrated network using various views [7]. By training these three algorithms, we attempt to investigate the optimal deep learning algorithm for detecting hepatic necrosis in non-clinical studies.

#### Model training

All procedures related to the algorithms’ training were performed using an open-source framework for machine learning (TensorFlow 2.1.0 using Keras 2.4.3 backend, and PyTorch) powered by an NVIDIA RTX 3090 24G GPU. Open-source packages for each algorithm (Mask R-CNN: torchvision [26], DeepLabV3 + : jfzhang95 pytorch-deeplab-xception package [27], SSD: amdegroot ssd.pytorch package [28]) were applied and their requirements were met in this study. Hyperparameters tuned for algorithm learning were adjusted accordingly (Online Resource 3) and each loss calculated according to the algorithm during the training was recorded and saved.

#### Loss

Loss in machine learning is the loss that occurs due to model estimation error when a learned model is applied to real data. Therefore, models with smaller losses offer a better prediction. In the case of object detection and segmentation for image analysis, various losses are calculated according to the type of algorithm. The total loss of Mask R-CNN is the sum of the classifier, box, mask, objectness, and region proposal network losses. The total loss of DeepLabV3^+^ is the result of calculating cross-entropy loss compared to the ground truth. In the case of SSD, localization loss and confidence loss are summed components of the total loss.

#### Metrics for model performance

**Fig. 2:**
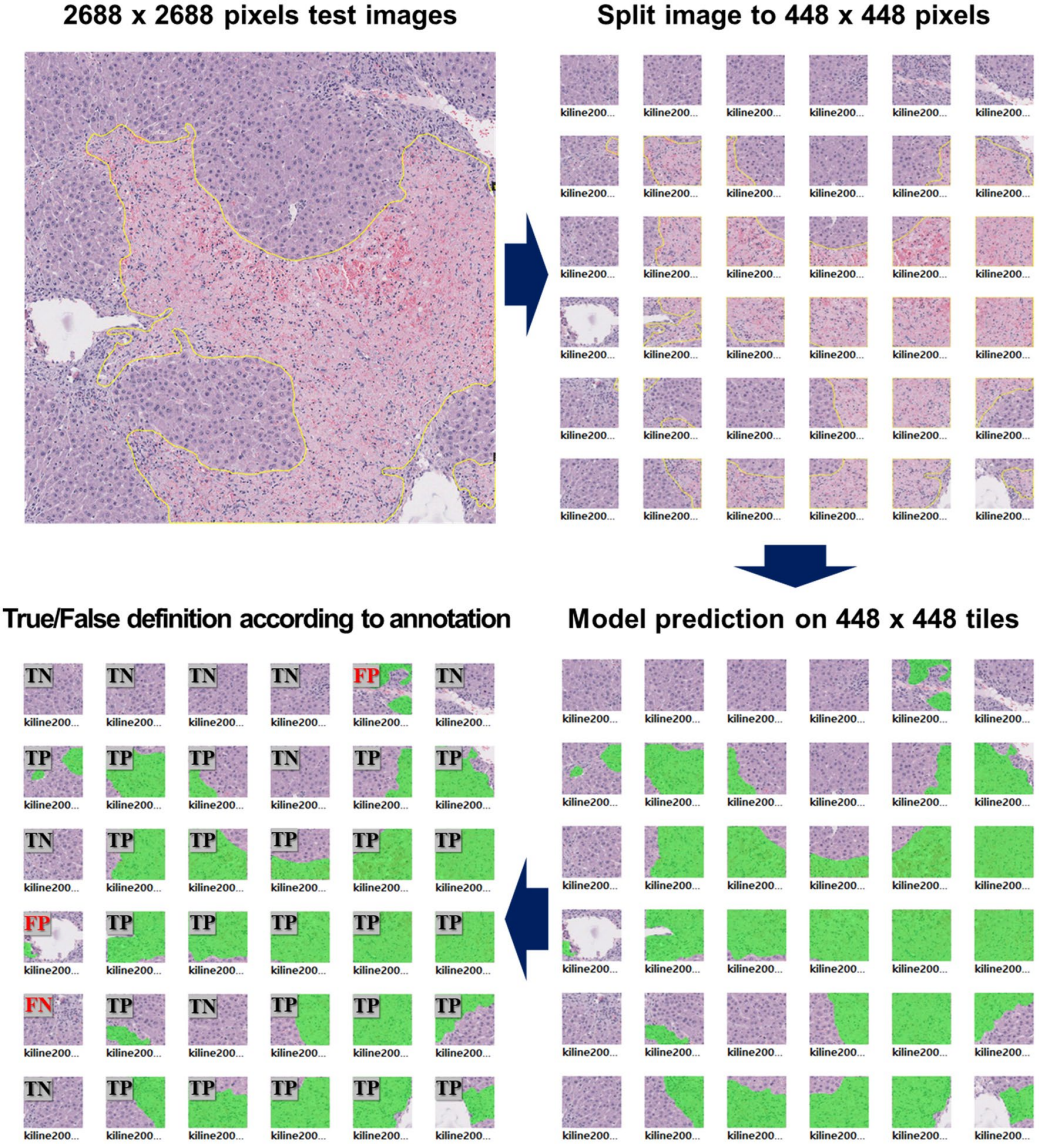
Procedure for calculating precision, recall, and accuracy values to evaluate each model’s performance in large-scale images. The annotated 2688 × 2688 images are split into 448 × 448-pixel tiles, and each model predicts the presence or absence of the lesion in each tile image. Subsequently, true and false predictions are defined according to the ground truth annotation, and the precision, accuracy, and recall values for each 2688 × 2688 image can be calculated

After training, each model calculates the mean intersection over union (IoU) by comparing the ground truth annotation to the predicted lesion according to each model’s trained weight from the test dataset. The IoUs calculated from the images in the test dataset were averaged and defined as the mean IoU. In the case of SSD, the method for calculating the mean IoU is different from that of the segmentation algorithms. The IoUs of SSD are defined as 1, 0.5, and 0.33 according to the prediction rates of 100%, 50%, and 33% of the number of predicted hepatic necrosis compared to the number of ground truth labels, respectively. Therefore, it is difficult to compare the performances of the three algorithms in terms of the mean IoU. To overcome this limitation and confirm the performance on large-scale images, we calculated and compared the precision, recall, and accuracy when predicting hepatic necrosis in 60 images (2688 × 2688 pixels) larger than the training images. Smaller 448 × 448-pixel tiles were derived from the larger 2688 × 2688 images. To calculate precision, accuracy, and recall values, the ground truth of the test images was annotated using the same procedure as when preparing the training data. The values were defined by the ratio of true positive, false positive, and false negative predictions according to the detected presence or absence of the lesion in each tile compared to the ground truth labels. A schematic diagram of the calculated precision, recall, and accuracy on the larger-scaled test images is depicted in Fig. 2, and the precision, recall, and accuracy are calculated by the following Eqs. (1–3). In addition, we calculated mask IoUs, which are IoUs from Mask R-CNN and DeepLabV3^+^, to confirm how precisely the models predicted the lesion area. The mask IoU is calculated by comparing the area of prediction to the ground truth annotations.

1 $${\text{Precision }} = \,\,\frac{TP}{{TP + FP}}$$

2 $${\text{Recall }} = \,\,\frac{TP}{{TP + FN}}$$

3 $${\text{Accuracy }} = \,\,\frac{TP + TN}{{TP + FN + FP + TN}}$$

## Results

### Algorithm training

**Fig. 3:**
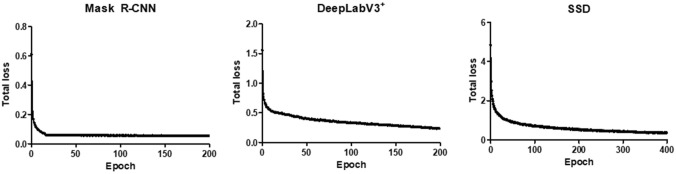
Total loss of each model calculated in every epoch during the training

To investigate the optimal deep learning algorithm for use in detecting hepatic necrosis in non-clinical studies, we trained three different algorithms, including Mask R-CNN, DeeplabV3^+^, and SSD, to detect hepatic necrosis. A total of 5,600 images with 5,680 annotations were used to train these three algorithms, and the total loss of each model was observed during training. Although the loss components calculated during the training varied between algorithms, the loss values for all three algorithms were quickly stabilized in the early phase of learning (Fig. 3). Therefore, each algorithm was successfully trained using the training dataset.

### Model accuracy, precision, and recall

**Fig. 4:**
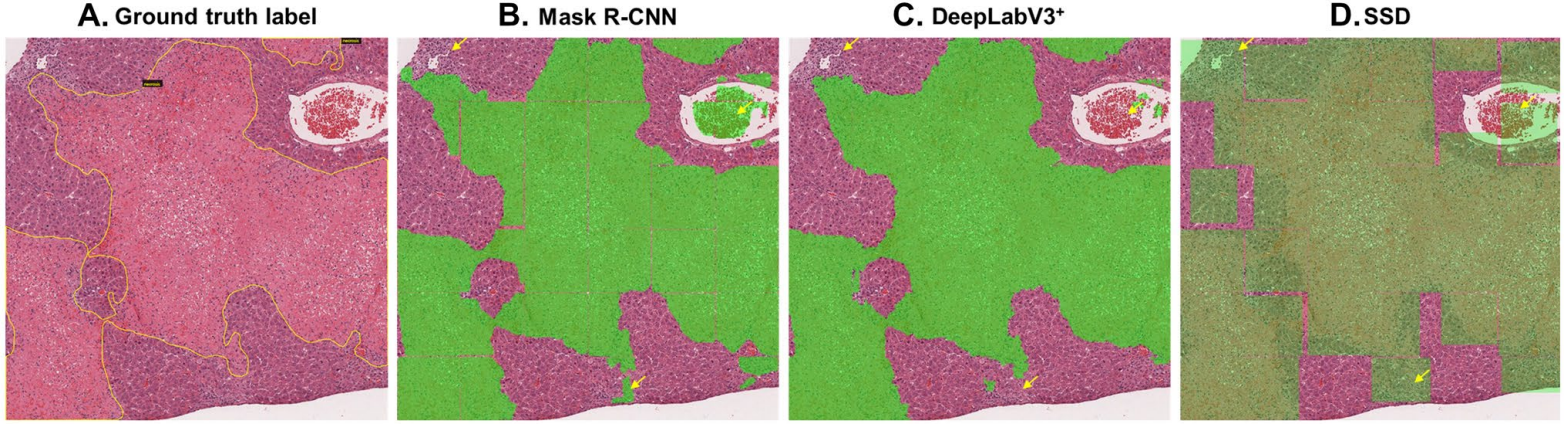
Prediction results by three trained models. Detected lesions on the image are indicated by green shading. Yellow arrows show the different detection errors of each model

After the model training, the mean IoU of each algorithm was calculated from the test dataset. The two segmentation algorithms showed a mean IoU of 0.94, suggesting that the predictions made were very similar to the ground truth annotations. The mean IoU of SSD was 0.99. However, due to the difference in the calculation methods of mean IoU between the segmentation algorithms and SSD, it is difficult to compare the performances of the three algorithms in this way. To compare the performance of each trained model and consider the implementation of the trained model for WSI-level analysis, we conducted additional tests with large-scale images. Each trained model predicted hepatic necrosis at the 448 × 448-pixel level within the 2688 × 2688 images. The results showed that all three algorithms successfully predict hepatic necrosis (Fig. 4). Notably, the trained Mask R-CNN model tends to not recognize the borderline of the 448 × 448-pixel tiles when the lesion occupies the entire tile. Furthermore, the trained SSD model’s prediction result includes not only the lesion but also normal cells due to its detecting method, the bbox. In some cases, the models incorrectly detected red blood cells (RBCs) and inflammatory cells as necrotic cells (yellow arrows in Fig. 4). However, DeepLabV3^+^ distinguished between these other cells and necrosis more consistently than the other models.

To evaluate the model’s performance mathematically, we calculated the precision, recall, and accuracy according to the ground truth label. True and false were preferentially defined in each 448 × 448-pixel tile according to the presence or absence of the predicted lesion compared to the ground truth. Then, the precision, recall, and accuracy were calculated according to the number of true positives, true negatives, false positives, and false negatives defined for each tile derived from the 60 test images (Table 1).

**Table 1:** Precision, recall, and accuracy calculated from large-scaled image prediction tests

	Precision	Recall	Accuracy
Mask R-CNN	0.94	0.94	0.92
DeepLabV3^+^	0.93	0.98	0.94
SSD	0.85	0.96	0.86

As a result, the performance of the two segmentation models, Mask R-CNN and DeepLabV3^+^, showed higher accuracy compared to that of the object detection model, SSD. Within segmentation models, the precision values were comparable. However, DeepLabV3^+^ showed the highest values in recall and accuracy of all the algorithms. The precision values indicate how many of the predictions the model makes are correct compared to the ground truth. Thus, the precision of the model prediction results for detecting hepatic necrosis was comparable between the two segmentation algorithms. Recall values show how close the predictions of hepatic necrosis by the trained model were to the ground truth. Therefore, the trained DeepLabV3^+^ showed good performance in distinguishing hepatic necrosis from other features in the image.

**Fig. 5:**
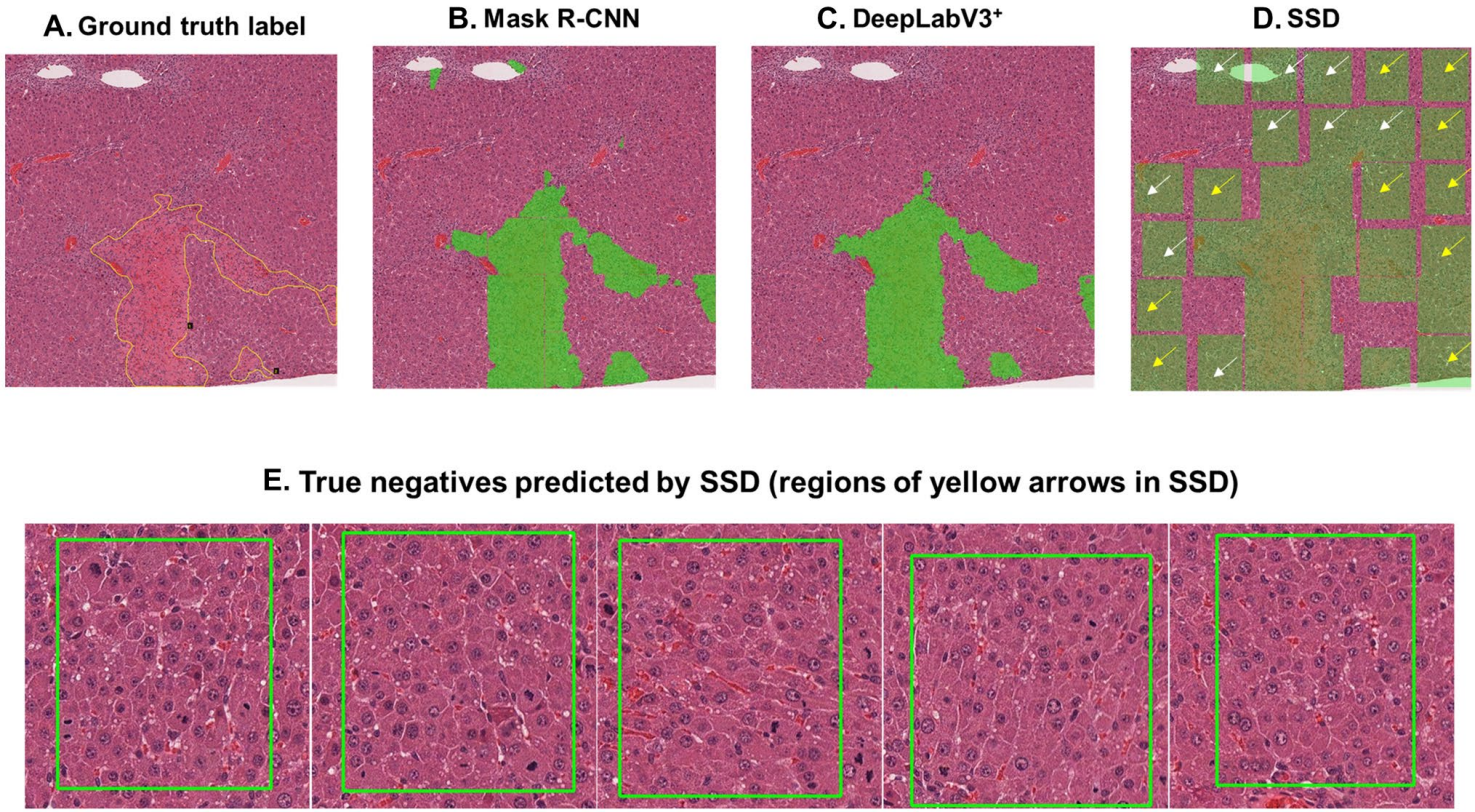
Incorrect prediction results of the SSD model (**d**) compared to the ground truth (**a**), Mask R-CNN (**b**), and DeepLabV3 + (**c**). Yellow arrows point to the normal cell regions incorrectly predicted as necrosis by SSD. The images of (**e**) are five examples of 448 × 448-pixel tiles from among the yellow arrows in (**d**). White arrows are pointing the inflammatory cells or RBCs that SSD incorrectly predicted as hepatic necrosis

In contrast, SSD, an object detection model, showed the lowest precision and accuracy of all the models. As shown in Fig. 4 and Fig. 5 (white arrows), the trained SSD frequently confused RBCs and inflammatory cells with necrotic cells. Moreover, in some cases, the trained SSD detected normal regions in the test images as hepatic necrosis (Fig. 5). These incorrect predictions contributed to its high false-positive rate of 10.9% compared to that of trained Mask R-CNN and DeepLabV3^+^ (4.3% and 4.1%, respectively). This is also reflected in the fact that SSD had the lowest precision value of all the models. Figure 5d shows the worst detection result, as predicted by the trained SSD.

When training, the SSD model learns all the image information included in the bbox annotation. Since the bbox label can only use square-shaped labels for annotation, it often includes normal cells and other features as well. In contrast, both segmentation models use a polygonal label that can separate hepatic necrosis from all other cells (Online Resource 1). The trained SSD annotation method leads to the incorrect prediction of normal liver cells, RBCs, and inflammatory cells as necrotic cells and is, therefore, not optimal for the task of detecting hepatic necrosis in non-clinical studies.

Precision and recall values only consider the presence or absence of the lesion in the test image tiles; therefore, details such as the area and exact location of the lesion are absent. Thus, we further calculated mask IoUs, for Mask R-CNN and DeepLabV3^+^ according to each prediction area compared to the ground truth annotations. As a result, the average mask IoUs of 0.75 for Mask R-CNN and 0.78 for DeepLabV3^+^ were observed. A mask IoU above 0.75 suggests that the area predicted by the trained algorithms as necrosis is comparable to the ground truth.

## Discussion

In this study, we trained three different algorithms, SSD, Mask R-CNN, and DeeplabV3^+^, for hepatic necrosis detection. A total of 5750 image tiles of 448 × 488 pixels with 5,835 annotations were successfully used to train, validation and test the algorithms, as demonstrated by the high values (> 0.94) of the mean IoU of each algorithm. However, it is difficult to compare the performances of the three algorithms in this way due to the differences in the calculation methods of the mean IoU. Therefore, we compared the performance of the models by calculating precision, recall, and accuracy values based on the prediction of 60 large-scaled images (2688 × 2688 pixels). As a result, two segmentation models, Mask R-CNN and DeepLabV3^+^, showed over 90% precision, accuracy, and recall values of model performance. Despite the prediction and labeling methods of these two algorithms being similar, Mask R-CNN and DeepLabV3^+^ are distinguished as instance segmentation and semantic segmentation models, respectively. On one hand, Mask R-CNN, a derivative of the Faster R-CNN, refers to the region proposal before the segmentation of the image, similar to object detection algorithms. Specifically, it first localizes the boxed ROI and then segments the object of interest by pixel. According to this procedure, the model segments every instance of the object of interest and recognizes them independently. On the other hand, DeepLabV3^+^ learns the object of interest at the pixel level, only discriminating whether the pixel is included in the object or not. We suggest that this variation in detection methods was reflected in the different precision and recall values of the two models and in the prediction results in Fig. 2, as DeepLabV3^+^ successfully distinguished between RBCs and inflammatory cells from hepatic necrosis. Therefore, the prediction results from the trained DeepLabV3^+^ for detecting the hepatic necrosis in test images are the closest to the ground truth label and show the highest accuracy among all the models. We suggest that this model is relatively reliable for detecting a single lesion of interest. Previous studies have shown nearly 100% accuracy in detecting hepatic necrosis when using a consolidated model trained with various other lesions [3, 17]. The accuracy values in this study are lower than those of previous research due to the false detection of other features such as inflammatory cells and RBCs as hepatic necrosis. The incorrect prediction observed in this study could be resolved by annotating cells that are often mistaken for lesions and including their exclusion in the training, together with the hepatic necrosis, as shown in our previous study [17].

In terms of precision and recall values, there might have been an imbalance between the two values. Therefore, researchers usually use the F1 score, a harmonic mean of two values, to compare the performance of the model [29]. In our study, the two segmentation models showed similar values in precision and recall, and the values of the trained SSD showed slight differences between them. This means that the data we used for the training phase were well-balanced and the algorithm training was successful. Overall, the model performance showed by the DeepLabV3^+^ makes it a reasonable single lesion detection model to be used in non-clinical studies.

Additional studies compared deep learning algorithms to find proper models for detecting a lesion of interest in a non-clinical study. Aubreville et al*.* compared segmentation, object detection, and regression models to analyze the mitotic count using canine cutaneous mast cell tumors. They found that a two-stage object detection model was comparable to and often outperformed veterinarians in detecting the most mitotically active tumor regions [30]. In contrast, the results of the present study on hepatic necrosis detection showed that the performances of the segmentation models were better than that of the object detection model. We hypothesize that this might be due to the large data sample size used in the previous study, whereas we used a small-scale in-house animal study. In addition, considering the characteristics of the lesion, necrosis has various components including bleeding, inflammatory cells, and dead cells not present during a mitotic count that and complicate lesion detection. Nevertheless, considering the F1 score (0.9) calculated from the precision and recall values, the trained SSD also showed good performance.

We examined three localization algorithms, Mask R-CNN, DeepLabV3^+^, and SSD, in terms of their detection methods and performance by calculating precision, recall, accuracy, and mask IoU. Most previous studies that applied localization deep learning algorithms to evaluate non-clinical safety and toxicologic pathologies used a single algorithm and compared its performance with that of veterinarians [11, 31–33]. Therefore, our research provides a comprehensive overview of the implementation of three deep learning algorithms for detecting a toxicologically significant lesion, hepatic necrosis. The two segmentation models outperformed the object detection algorithm, SSD, when distinguishing the lesion from other features in the test data. All algorithms, including SSD, showed good performance (over 85% accuracy) for the detection of hepatic necrosis. In addition, using the mask IoU, we confirmed that the accuracy of the predictions of Mask R-CNN and DeepLabV3^+^ were similar to the ground truth in the large-scale test images. Indeed, the Mask R-CNN, as an instance segmentation model, considers not only the IoU, but also the number of predicted objects compared to the number of ground truth annotations when calculating the model accuracy. If the model predicted the area of lesion of interest correctly, but the number of predictions is different from that of the annotation, this can be considered an incorrect prediction that lowers the accuracy. However, we argue that it is more important to predict the exact area of the lesion of interest than the number of predicted instances of the lesion within an image. Thus, we suggest that using mask IoU, calculated according to each prediction area compared to the ground truth annotations, could be useful when evaluating the model performance in the prediction of a lesion of interest in non-clinical studies.

In conclusion, it is important to determine the potentially confusing components within the images and implement this in the training of the algorithm. A comprehensive understanding of the characteristics of the used algorithm is essential for detecting a lesion of interest. Overall, a segmentation algorithm might be a proper algorithm for pathological analysis in non-clinical studies. The segmentation methods could give more intuitive and numerical statistical information to users in terms of the visualization of a prediction. Therefore, we expect that the application of the segmentation algorithm to non-clinical studies would contribute to bringing a more evident and visualized decision to the evaluation of the toxicity of the test item.

## Supplementary Information

Below is the link to the electronic supplementary material.

Supplementary file1 (PDF 262 KB)

## Data Availability

The datasets generated and/or analyzed during the current study are not publicly available due to their being currently under examination for copyright registration but are available from the corresponding author upon reasonable request.
