# Supplementary material for: A comparative study on the implementation of deep learning algorithms for detection of hepatic necrosis in toxicity studies
Source: Toxicol Res. 2023 Apr 6;39(3):399–408. doi: 10.1007/s43188-023-00173-5 (PMC10313597; doi:10.1007/s43188-023-00173-5)
Supplement: Supplementary file 1 — Supplementary file1 (PDF 262 KB) [file 43188_2023_173_MOESM1_ESM.pdf]

Supplementary Information

A comparative study on the implementation of deep learning algorithms for detection of hepatic necrosis in toxicity studies

Journal: ***Toxicological Research***

Ji-Hee Hwang, Minyoung Lim, Gyeongjin Han, Heejin Park, Yong-Bum Kim, Jinseok Park, Sang-Yeop Jun, Jaeku Lee, Jae-Woo Cho

[1]Toxicologic Pathology Research Group, Department of Advanced Toxicology Research, Korea Institute of Toxicology, Daejeon 34114, Korea

[2]Department of Advanced Toxicology Research, Korea Institute of Toxicology, Daejeon 34114, Korea

[3]Research & Development Team, LAC Inc., Seoul 07807, Korea

*Correspondence: cjwoo@kitox.re.kr; Tel.: +82-42-610-8023

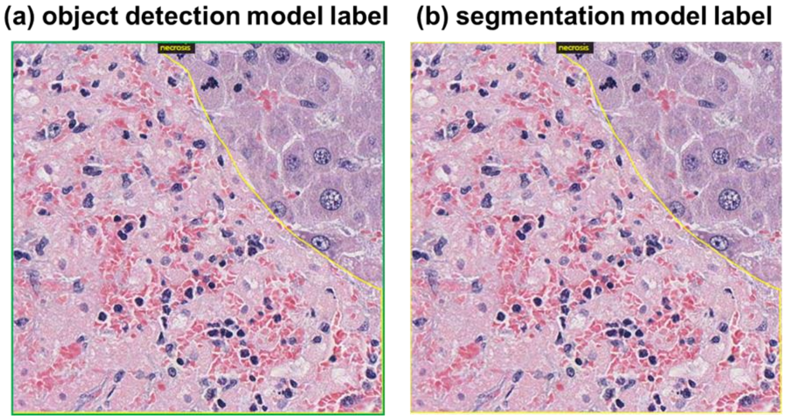

Online Resource 1 - The examples of labels (see yellow outline) according to the models. The lesions were characterized using nuclear dissolution and fragmentation with pale eosinophilic cytoplasm in the image and hemorrhage. In addition, the object detection model SSD's label includes not only the hepatic necrosis but also normal cells in the bounding box annotation (a), occasionally making the 448 × 448-pixel tile itself the annotation for the hepatic necrosis (see green outline). The segmentation model separates the lesion from other cells in the annotation (b).

Online Resource 2. The number of images and annotations used for algorithm training. The numbers in parentheses include augmented images or annotations created by reverse, rotation, and brightness image-augmenting techniques.

	Number of images	Number of annotations
**Train**	350 (5600)	355 (5680)

**Validation**	100	104
**Test**	50	51
**Total**	500 (5750)	510 (5835)

Online Resource 3. Hyperparameters used for training each network

Network Type	Mask R-CNN	DeeplabV3$^+$	SSD
**Parameters**	BACKBONE = resnet152	BACKBONE = "resnet"	COCO_API = "PythonAPI"
	DIST_BACKEND = "nccl"	LR = 0.01	EPOCHS = 400
	BATCH_SIZE = 16	EPOCHS = 200	BATCH_SIZE = 64
	LR = 0.005	BATCH_SIZE = 32	LR = 0.0003
	EPOCHS = 200	DATASET = "COCO"	WEIGHT_DECAY = 0.00003
	WORKERS = 16	MASK_THRESHOLD = 0.6	MOMENTUM = 0.9
	LR_STEPS = 16, 22		CONFIDENCE = 0.5
	CONFIDENCE = 0.5		
